# Research on Decoupling Model of Six-Component Force Sensor Based on Artificial Neural Network and Polynomial Regression

**DOI:** 10.3390/s24092698

**Published:** 2024-04-24

**Authors:** Shuyu Wang, Hongyue Liu

**Affiliations:** School of Mechatronic Engineering and Automation, Shanghai University, Shanghai 200444, China

**Keywords:** six-component force sensor, decoupling, back propagation neural network, polynomial fitting, fiber Bragg grating sensor

## Abstract

A two-stage decoupling model based on an artificial neural network with polynomial regression is proposed for the six-component force sensor load decoupling problem in the case of multidimensional mixed loading. The six-dimensional load categorization stage model constructed in the first stage combines 63 load category label sets with a deep BP neural network. The six-dimensional load regression stage model was constructed by combining polynomial regression with a BP neural network in the second stage. Meanwhile, the six-component force sensor with a fiber Bragg grating (FBG) sensor as the sensitive element was designed, and the elastomer simulation and calibration experimental dataset was established to realize the validation of the two-stage decoupling model. The results based on the simulation data show that the accuracy of the classification stage is 93.65%. The MAPE for the force channel in the regression stage is 6.29%, and 3.24% for the moment channel. The results based on experimental data show that the accuracy of the classification stage is 87.80%. The MAPE for the force channel in the regression phase is 5.63%, and 4.82% for the moment channel.

## 1. Introduction

With the rapid development of rotorcraft such as UAVs and helicopters, six-component force sensors and fiber Bragg grating sensors have become increasingly important in the field of aerospace structure monitoring [1,2,3,4,5,6,7,8], which can simultaneously measure the mechanical quantities $F_{x},F_{y},\;F_{z},M_{x},M_{y},M_{z}$ of six degrees of freedom in space. In practical applications, there is cross sensitivity among the measurement dimensions of the six-component force sensor; that is, in the case of multidimensional mixed loading, the output signal of a single dimension of the sensor will be affected by other dimensions. The decoupling model separates these effects through mathematical methods to improve the output accuracy and application generalization of the sensor [9,10,11,12,13,14,15]. Therefore, the decoupling model optimization of the six-component force sensor is very important in the field of rotor structure monitoring.

Current decoupling models can be divided into linear and nonlinear models. In 1978, B.E. Semino et al. [16] used linear regression for the first time to decouple a four-beam six-component force sensor. In 1995, Patra JC et al. [17] used a BP neural network for the first time to decouple pressure sensors. In 2002, Jiang Li et al. [18] used a BP neural network to decouple a micro-fingertip sensor, and the experimental results showed that the decoupling accuracy was higher than that in linear regression. In 2013, Gai Guanghong et al. [19] took a Stewart six-component force sensor as the research object and proposed an averaging linear decoupling matrix to reduce the degree of error fluctuation. In 2016, Zhang Jiamin et al. [20] trained the decoupling model of a BP neural network using single-dimensional loading calibration experimental data, analyzed the decoupling performance of model training under a different number of neurons in hidden layers, and conducted experimental verification. In 2018, Yingjun Li et al. [21] proposed a decoupling model combining linear regression and support vector machine regression, and conducted decoupling experiments. In 2020, Xu Jiaqi et al. [22] proposed a stochastic forest decoupling model optimized by a genetic algorithm. Experimental results show that the operation time of this model is shorter and the accuracy is improved on the basis of ordinary stochastic forests. In 2023, Wang Zhijun et al. [23] proposed a decoupling model based on polynomial regression and conducted calibration experiments to verify the conclusion that decoupling error is better than linear regression. For nonlinear models, a BP neural network, random forest, and so on are widely used. In 2023, Zha Hao et al. [24] developed an online decoupling module of a BP neural network optimized by a genetic algorithm based on EtherCAT (bus communication system) and carried out field data acquisition, and the results showed that the decoupling accuracy was better than that in linear regression. The above research mainly analyzes the static decoupling problem of sensors under single-dimensional loading. For a linear model, this model is simple and only needs single-dimensional calibration data to achieve decoupling, but the accuracy and generalization are low, and a linear model is more suitable for load decoupling under integer multiple-step loading. For nonlinear models, the accuracy is generally better than that in linear models, but more data are needed to build the model. To sum up, although the sensor decoupling model has been widely studied, there are few studies on decoupling under multidimensional mixed loading in practical engineering.

To solve these problems, a two-stage decoupling model combining linear and nonlinear is proposed. The first stage of the model is load classification, and the second stage is load regression. At the same time, the elastomer structure and sensitive element layout scheme are designed, the elastomer is simulated by ANSYS, and the training set and verification set are made based on the sensitive element layout position. Finally, MATLAB and a simulation dataset are used to construct a two-stage decoupling model and verify the model performance. It provides a research idea for promoting the technical progress of a six-component force sensor in a space structure monitoring field.

## 2. Design of Sensor

Due to the advantages of small size, electromagnetic interference resistance, and high sensitivity of a fiber Bragg grating sensor (hereinafter referred to as FBG), the overall scheme of a six-component force sensor is designed based on FBG and a load-bearing beam elastomer. The design steps include the following: the elastomer and assembly structure of the six-component force sensor was designed, the FBG layout scheme was designed, and the decoupling model was established based on the elastomer structure and FBG sensing principle. Details are as follows.

### 2.1. Structural Design of Sensor Elastomer and Assembly

**Figure 1:**
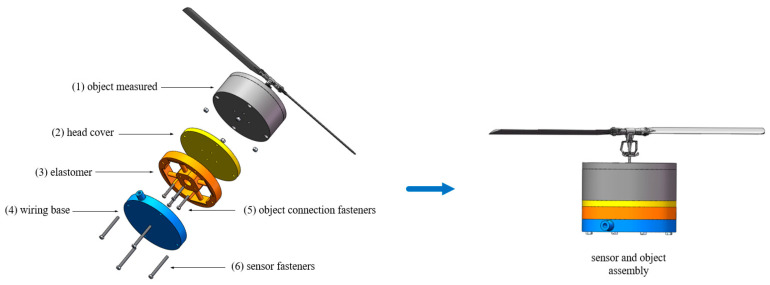
Six-component force sensor and object measured.

The designed six-component force sensor is shown in Figure 1, and the overall size is 300 mm × 300 mm × 100 mm. The main structure of the sensor includes (1) the measured object, (2) the top cover, (3) the elastomer, (4) the wiring bottom cover, (5) the fastener for the measured object, and (6) the sensor fastener. The measured object in Figure 1 is the double-blade rotor and the motor module. (5) The connected fasteners of the object to be measured connect the elastomer, the top cover, and the object to be measured by bolts, and the load generated when the object to be measured works is transmitted to the center table through bolts and then transmitted to the four bearing beams, which are sensed by the sensitive elements. (3) Cylindrical beams are selected for elastomer bearing beams because, compared with rectangular beams, the bending section coefficient of circular sections under the same size conditions is smaller, and the structure is more sensitive to strain changes. Thin-walled floating beams around the perimeter amplify the strain. (6) Sensor fasteners assemble the sensor as a whole through bolts and nuts.

### 2.2. FBG Sensing Principle and Layout

**Figure 2:**
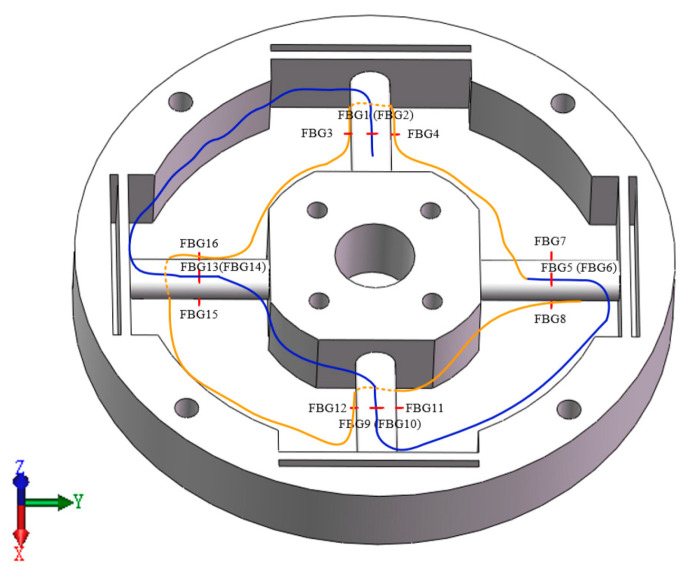
Elastomer structure and placement location of FBG sensors.

As shown in Figure 2, the six-component force sensor uses 16 FBG as the sensitive element, including eight groups of two in each group. Each group is symmetrical about the cylinder and pasted on the surface of the elastomer along the length of the cylinder beam. The position of the grid area is the midpoint along the length of the cylinder beam. When $F_{x}$ is applied, FBG15 and FBG16 are a set of differential symmetric positions, one stretching, the other compressing; FBG8 and FBG7 are a set of differential symmetric positions, one is stretched, the other is compressed, and the two positions of each group produce equal and opposite signs of strain, which can realize signal amplification and temperature compensation after subtraction. For other positions, for instance, FBG1 and FBG2 are both stretched, and FBG11 and FBG12 are compressed and are zero after subtraction, which has no effect on $F_{x}$. Therefore, only FBG15, FBG16, FBG8, and FBG7 are included in the $F_{x}$ calculation relation. The calculation logic of the load is consistent with that of $F_{x}$.

**Figure 3:**
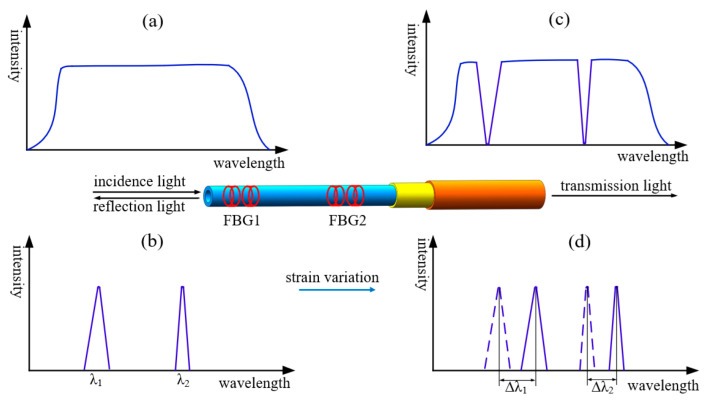
Principle of FBG sensing: (**a**) incidence spectrum, (**b**) reflection spectrum, (**c**) transmission spectrum, and (**d**) reflection spectrum after the center wavelength shift.

The FBG sensing principle [25] is shown in Figure 3, a six-component force sensor decoupling model is established based on this principle, and the feasibility of using a neural network and other models for multidimensional load decoupling is illustrated. When the light enters the core and passes through the gate area, reflection and transmission occur, and the light that meets the specific wavelength will be reflected; the specific wavelength is the central wavelength. The central wavelength satisfies the following relation:

(1) $$\lambda =2n_{eff}\Lambda$$

where λ is the central wavelength of FBG, $n_{eff}\;$ is the effective refractive index of the fiber core, and Λ is the grating period.

When there are stress and temperature changes around the fiber, the central wavelength will shift, as shown in Figure 3d,

(2) $$\Delta \lambda =\lambda \left[\left(1-P_{e}\right)\Delta \varepsilon +\left(\alpha +\xi \right)\Delta T\right]$$

where Δλ is the change in central wavelength, Δε is the change in strain, and ΔT is the change in temperature. For quartz FBG at room temperature, the $P_{e}$ elasto-optical coefficient is 0.22, the α thermal expansion coefficient is $5.5\times 10^{-7}/℃$, and the ξ thermo-optical coefficient is $6.8\times 10^{-6}/℃$.

Taking the symmetric measurement points FBG1 and FBG2 in the same temperature field as an example, the center wavelength changes of these two measurement points are $\Delta \lambda_{1}$ and $\Delta \lambda_{2}$, respectively, and the strain changes are $\Delta \varepsilon_{1}\;$ and $\Delta \varepsilon_{2}$, and the center wavelength shift changes are obtained by differential operation as follows:

(3) $$\Delta \lambda_{1}-\Delta \lambda_{2}=\lambda \left(1-P_{e}\right)\left(\Delta \varepsilon_{1}-\Delta \varepsilon_{2}\right)$$

### 2.3. Establishment of Decoupling Model

According to Equation (3), the temperature-compensated FBG center wavelength versus strain is defined as follows:

(4) $$E_{r}=a_{1}\Lambda$$

(5) $${\left(\Delta \varepsilon_{1}-\Delta \varepsilon_{2},\Delta \varepsilon_{4}-\Delta \varepsilon_{3},\ldots ,\Delta \varepsilon_{15}-\Delta \varepsilon_{16}\right)}^{T}=a_{1}{\left(\Delta \lambda_{1}-\Delta \lambda_{2},\Delta \lambda_{4}-\Delta \lambda_{3},\ldots ,\Delta \lambda_{15}-\Delta \lambda_{16}\right)}^{T}$$

In Equation (4), $E_{r}$ is the change of eight groups of strain, Λ is the change of eight groups of central wavelength, and $a_{1}=\frac{1}{\lambda (1-P_{e})}$ is the conversion coefficient between the change of strain and central wavelength. In Equation (5), the subscript of strain and wavelength is the serial number of the measuring point position.

Define the relationship between stress and strain as follows:

(6) $$F_{r}=a_{2}E_{r}$$

(7) $$F_{r}={\left(f_{1}-f_{2},\;f_{4}-f_{3},\ldots ,f_{15}-f_{16}\right)}^{T}$$

In Equation (6), $F_{r}$ is the stress variation of eight groups, and $a_{2}$ is the stress–strain conversion coefficient. In Equation (7), the subscript of stress is the position sequence number.

Combining the elastomer and the FBG arrangement position defines the load matrix $L_{d}$ as follows:

(8) $$L_{d}=\left(\begin{array}{c} F_{x}\\ F_{y}\\ F_{z}\\ M_{x}\\ M_{y}\\ M_{z} \end{array}\right)=\left(\begin{array}{c} k_{1}\left[\left(f_{15}-f_{16}\right)+\left(f_{8}-f_{7}\right)\right]\\ k_{2}\left[\left(f_{11}-f_{12}\right)+\left(f_{4}-f_{3}\right)\right]\\ k_{3}\left[\left(f_{1}-f_{2}\right)+\left(f_{5}-f_{6}\right)+\left(f_{9}-f_{10}\right)+\left(f_{13}-f_{14}\right)\right]\\ k_{4}r\left[\left(f_{5}-f_{6}\right)+\left(f_{14}-f_{13}\right)\right]\\ k_{5}r\left[\left(f_{10}-f_{9}\right)+\left(f_{1}-f_{2}\right)\right]\\ k_{6}r\left[\left(f_{3}-f_{4}\right)+\left(f_{7}-f_{8}\right)+\left(f_{11}-f_{12}\right)+\left(f_{15}-f_{16}\right)\right] \end{array}\right)$$

$k_{i\;}$is the conversion coefficient of the change in the i load and stress, and r is the vertical distance from the measuring point to the center of moment of the elastomer. This formula shows that Equation (8) is obtained by matrix decomposition.

(9) $$L_{d}=G_{1}G_{2}F_{r}$$

$G_{1}$ and $G_{2}$ are the coefficients’ matrix after decomposition. Since the simulation strain data are used in this paper for model verification, Equation (6) is brought into Equation (9) to obtain the following:

(10) $$L_{d}=a_{2}G_{1}G_{2}E_{r}$$

Let $U=a_{2}G_{1}G_{2}$.

(11) $$L_{d}=UE_{r}$$

From Equation (11), it can be seen that $L_{d}$ is the output matrix of model training, which corresponds to the six-dimensional load values set in the simulation; $E_{r}$ is the input matrix, which corresponds to the eight sets of FBG strain variations or the simulation strain results; U is the model parameter matrix for the linear model; and U is the model weight for the nonlinear model such as the BP neural network, which further shows that the numerical relationship between the FBG strain data and the six-dimensional load can be represented by models such as the BP neural network and other models.

## 3. Decoupling Model Theory

On the basis of sensor design, the proposed two-stage decoupling model is further discussed. The discussion of the model includes describing the overall framework of the model, explaining the content and establishment method of the model dataset, and analyzing the comparison results and evaluation indicators between the other four models and the two-stage model, and the specific contents are as follows.

### 3.1. Definition of Load Category Label

The definition of the load category label is shown in Table 1. The six-bit binary number and the corresponding decimal number are used to define the payload category. When the payload exists, the corresponding bit is assigned to 1. When the load does not exist, the value is 0, and since the load has six dimensions, there are $2^{6}-1$ categories excluding the case of no load. For example, when $F_{x},F_{y},F_{z}$ exists and $M_{x},M_{y},M_{z}$ does not exist, the binary label is (1,1,1,0,0,0) and the corresponding decimal label is 56. It is important to note that the decimal label acts as the input and output of the classification neural network. The above definition method can effectively quantify the load categories and be applied to the operation of neural networks.

### 3.2. Framework of Decoupling Model

**Figure 4:**
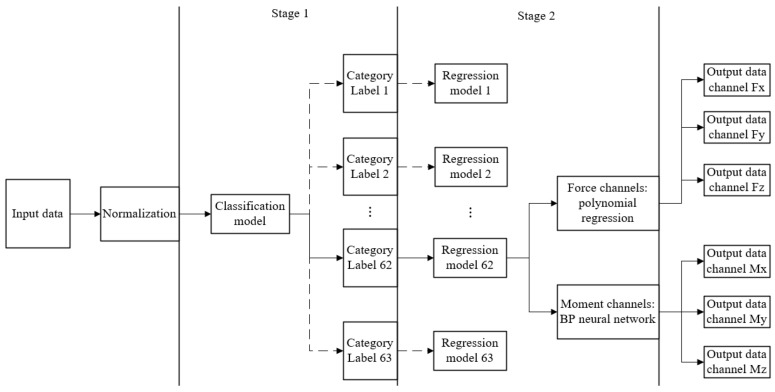
Framework of decoupling model.

The framework of the six-component force sensor decoupling model is shown in Figure 4. The model mainly consists of two stages. The first stage (stage 1) is the classification stage. The second stage (stage 2) is the regression stage, which applies the corresponding regression model for different load categories and output channels to obtain the load value corresponding to the strain data.

The solid line in Figure 4 represents the model running route when the classification model recognizes the strain data as category label 62. The running route is used as an example to introduce the model framework in detail.

(a) Preprocessing of input data

The preprocessing is the normalization of strain data, and all eight strain data are mapped to values in the range of 0 to 1, mainly for the training process and verification process of neural networks, with the purpose of speeding up the convergence speed of network training and preventing overfitting.

(b) Classification stage of model

The preprocessed strain data are input into the neural network classification model, and the decimal class label 62 is obtained first, and then the decimal label is converted into the six-bit binary label 111110. For the channel $M_{z}$ with binary bit 0, its regression value is directly set to zero.

(c) Regression stage of model

For the channel whose binary label bit is not 0, the corresponding regression model is used to carry out load value regression. Polynomial regression is used for the first three $F_{x},F_{y},$ and $F_{z}$ channels, and a BP neural network is used for the last three $M_{x},M_{y}$, and $M_{z}$ channels. In particular, there are regression models for each output channel of each category of force labels; for example, the polynomial regression model for the $F_{x}$ channel of label 62 is qt_regression_62_1; there are regression models for each category of torque labels. For example, the BP neural network regression model corresponding to label 62 is bp_regression_62. Additionally, the training set of the regression model is the corresponding class of data, rather than all the data. Based on the verification set regression results of a single model (no classification stage), the polynomial regression error for the force channel is small, and the BP neural network regression error for the torque channel is small. Therefore, the combination of a BP neural network and polynomial regression is selected for the regression stage.

(d) Outputting result

The regression values of all six channels are summarized and output.

### 3.3. Theory of Classification Stage

**Figure 5:**
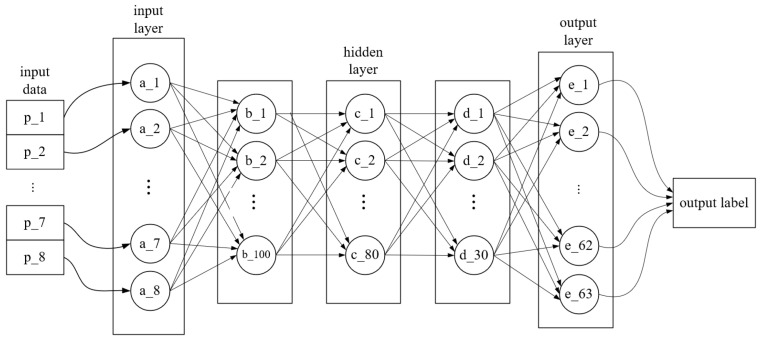
Classification stage network model.

Since the zero-setting operation and regression model selection in the model depend on the accuracy of classification, the classification stage is the key to affect the accuracy of the model. The decimal class label and eight strain data are input into the classification-stage deep BP neural network to obtain the corresponding load decimal class label. The network structure of this stage is shown in Figure 5.

The input data in the Figure 5 is the normalized strain value vector $P={\left(p_{1},p_{2},\ldots ,p_{8}\right)}^{T}$. $A={\left(a_{1},a_{2},\ldots ,a_{8}\right)}^{T}$ is the input layer vectors. The number of neurons in the three hidden layers is 100-80-30. ReLU is the hidden layer activation function. A Softmax layer is used for the tag probability mapping layer behind the hidden layer. $E={\left(e_{1},e_{2},\ldots ,e_{63}\right)}^{T}$ is the output layer neuron, corresponding to the probability of 63 load classes, and the class label corresponding to the maximum probability in E is taken as the final output in the classification stage. The number of iterations was 2000, and the learning rate was 0.05.

Since the classification stage is very important for this decoupled model, which is associated with the zero-setting operation, the selection of the corresponding regression model operation, the requirement of high prediction accuracy, and the high number of output categories (63), it was chosen to construct a three-layer deep BP neural network with a high number of neurons using the trainNetwork function in MATLAB to fit the relationship between the load values and the load categories. Generally, the settings of the number of neurons and hidden layers for a particular task are based on experience and repeated testing, for example, first selecting a smaller number of hidden layers, selecting a number of neurons that are multiplicatively related to the dimensionality of the input data, increasing or decreasing the number and testing it continuously to observe the prediction performance of each setting, and finally, picking out the most appropriate number of neurons and hidden layers. The decoupling task in this paper requires less data to be processed compared with the image processing task; therefore, initially, three hidden layers (a neural network with at least three hidden layers is known as a deep neural network) and close to two times the number of output dimensions (126) are selected as the number of neurons for the first hidden layer, and then the number of neurons is continuously increased or decreased and tested in steps of 10 to observe the prediction accuracy. Finally, 100-80-30 is determined as the hidden layer setup scheme with the highest accuracy.

### 3.4. Theory of Regression Stage

After obtaining the load category label and zeroing the channel with binary label bit 0, eight strain data are input into the polynomial model in the regression stage, and the regression values of the three force channels are obtained. The polynomial used for regression is Equation (12). The $m_{i},\;n_{ij},\;k_{i}$ in the polynomial respectively represent the coefficient of the first term, product term coefficient, and square term coefficient; c represents the constant term. Polynomial coefficients are solved based on MATLAB’s fitlm function, whose fitting model parameter is quadratic.

(12) $$f\left(p_{1},p_{2},\ldots ,p_{8}\right)=\sum_{i=1}^{8}m_{i}p_{i}+\sum_{i=1}^{7}\sum_{j=i+1}^{8}n_{ij}p_{i}p_{j}+\sum_{i=1}^{8}k_{i}{p_{i}}^{2}+c$$

Taking the $F_{x}$ channel of label 41 as an example, its coefficients and constant terms are shown in Table 2. If no item appears in the table, its coefficient is 0.

**Figure 6:**
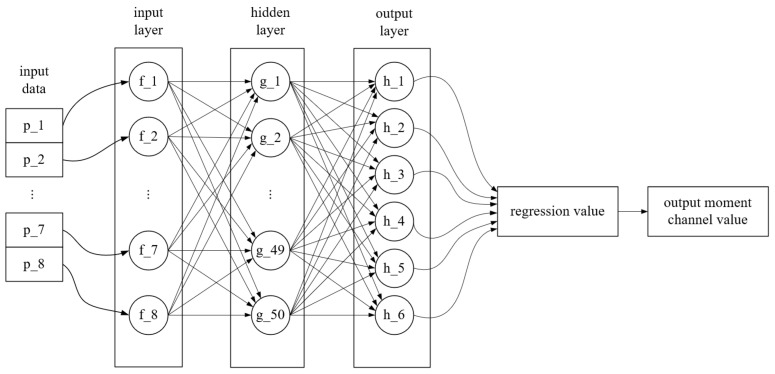
Network model of force channel in regression stage.

Eight strain data are input into the BP neural network in the regression stage, the regression value of the six-dimensional load is obtained, and the results of the three moments of the force are output. The network structure of this stage is shown in Figure 6.

The input data in Figure 6 are consistent with the classification model, which are strain value vectors $P={\left(p_{1},p_{2},\ldots ,p_{8}\right)}^{T}$. The hidden layer is the fully connected layer of 50 neurons. $H={\left(h_{1},h_{2},\ldots ,h_{6}\right)}^{T}$ is the output layer neuron, corresponding to the load values of six load channels, but only outputting the regression values of three torque channels. The number of training iterations is 2000, and the learning rate is 0.001. Finally, the regression values of the three force channels and three moment channels are summarized to form the final six-dimensional load output.

Since the input dimension (8) and output dimension (6) of the network in the regression stage are relatively small, the feedforwardnet function was initially chosen to construct a neural network with a single hidden layer and two times the number of hidden layers in the input dimension (16), and then the number of neurons was increased or decreased and tested in steps of 5. Finally, a single layer and 50 neurons were determined to be the setup solution with the smallest prediction error.

## 4. Simulation Verification and Analysis of Results

### 4.1. Simulation Setup and Establishment of Dataset

**Figure 7:**
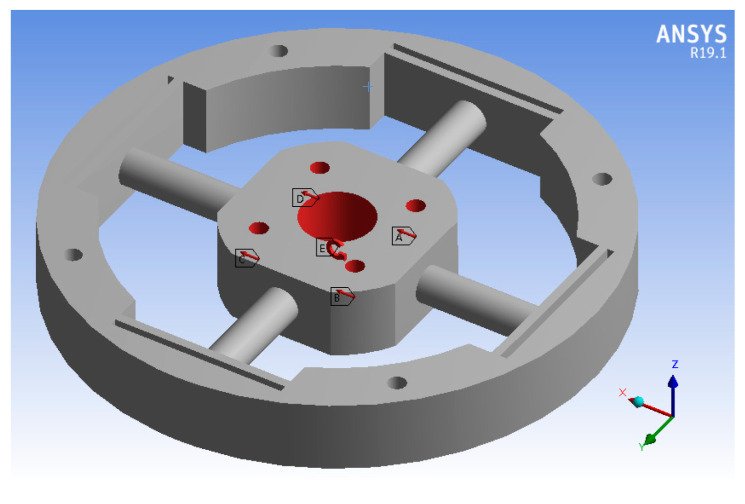
Simulation load setting.

**Figure 8:**
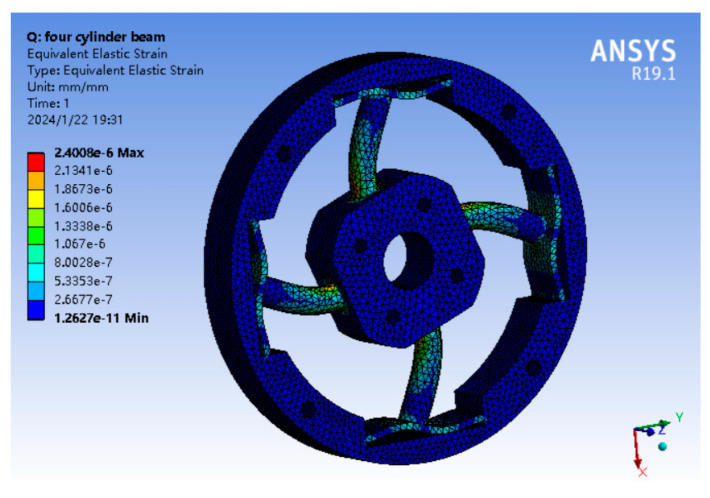
Simulation result.

In order to ensure the generalization of the neural network model, the random number generation program is used to generate the values of force and moment as the simulation load data of the elastomer, where the force load ranges from 1000 N to 10,000 N, and the torque load ranges from $1\times 10^{5}$ to $1\times 10^{6}\;$N·mm. As shown in Figure 7, the force load is evenly loaded in the geometric center of the four bolt holes of the center table, and the torque is loaded in the geometric center of the central through hole. The elastomer material is aluminum alloy. The outer wall of elastomer is a fixed constraint. The strain extraction location is the 16 FBG locations in Figure 2, and the strain data are extracted according to the node ID to ensure that the collection location of each group of data is completely consistent. After exporting all the strain data, it is converted into eight-dimensional data according to the differential symmetric position. The simulation results when $M_{z}$ is loaded are shown in Figure 8. The total number of samples in the training set is 3141, in which the number of samples loaded in a single dimension is 20, the number of samples loaded in a double dimension is 30, and the number of samples from three to six dimensions is 60. The total number of samples of the classification verification set is 63. The total number of samples of the regression verification set is 117.

After the simulation results are exported, the decoupling model dataset is made, as shown in Table 3. Label is the label of the load category. Force and moment are six-dimensional load values. Strain is the eight-dimensional simulated strain value after a differential budget.

### 4.2. Result of Classification Stage Based on Simulation Data

**Figure 9:**
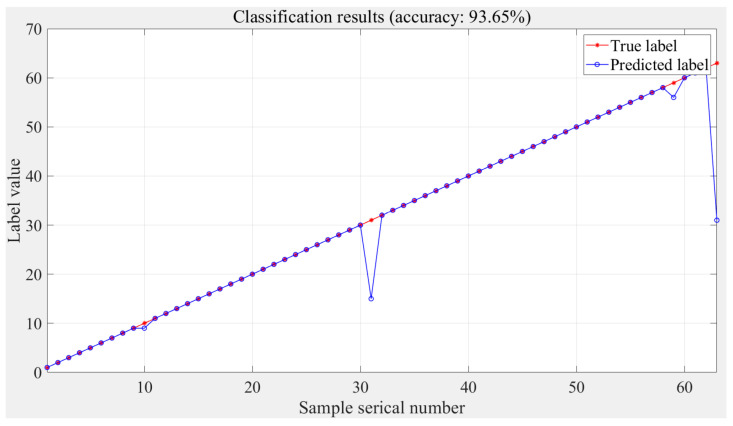
Classification stage results based on simulation data.

The prediction results of the classification stage based on the simulation data are shown in Figure 9, with an accuracy of 93.65% after 2000 generations of training. The horizontal coordinate is the validation set sample serial number, and the vertical coordinate is the label value., where red is the true label of the validation set and blue is the predicted label. The labels with errors are 10, 31, 59 and 63, and after converting the decimal labels to binary, it can be seen that the model has relatively poor prediction performance for multidimensional loads.

### 4.3. Result of Regression Stage Based on Simulation Data

**Figure 10:**
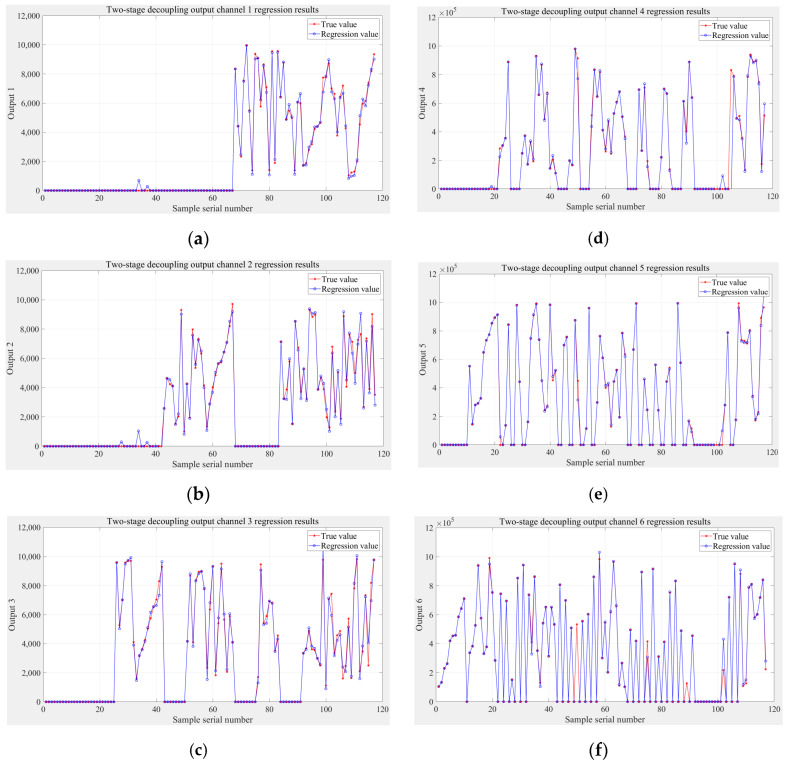
Regression stage results based on simulation data: (**a**) $F_{x}$, (**b**) $F_{y}$, (**c**) $F_{z}$, (**d**) $M_{x}$, (**e**) $M_{y}$, (**f**) $M_{z}.$

The results of the regression stage based on the simulation data are shown in Figure 10, which shows the regression of the six-dimensional load in the verification set, where the training algebra of the torque channel neural network is 2000. The horizontal coordinate is the sample number of the verification set, and the vertical coordinate is the load value. The blue line is the load regression value, and the red line is the true load value of the verification set. As can be seen from Figure 10 as a whole, under the action of prediction and zero-setting in the classification stage, the two-stage model has a good regression effect on the 0 values of the six channels, which reflects the importance of the classification stage in this model. By comparing the subgraphs of a force channel and a moment channel, it can be seen that the regression result of the two-stage model is better than that of the force channel. Combining the prediction results of the classification stage (the label of prediction error) and the data points with large errors in each subgraph, it can be seen that the regression performance of the two-stage model for multidimensional loads is slightly poor, and there is still room for optimization in training sets and network parameters.

### 4.4. Analysis of Model Comparison

Four single models are selected for comparison, which are linear regression, polynomial regression, random forest, and BP neural network. The single model here refers to the nonclassification stage.

**Figure 11:**
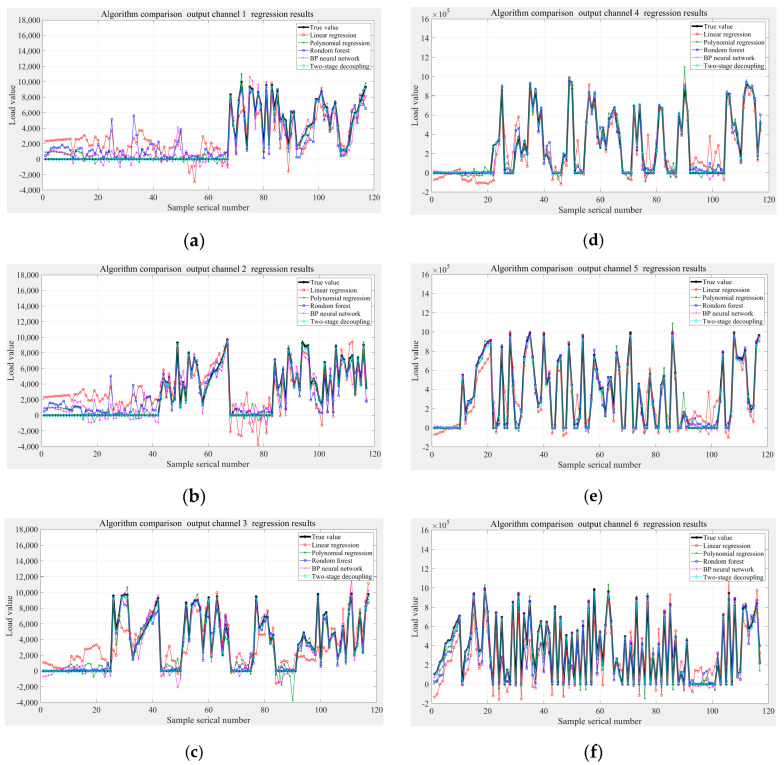
Regression results comparison of five models based on the simulation data: (**a**) $F_{x}$, (**b**) $F_{y}$, (**c**) $F_{z}$, (**d**) $M_{x}$, (**e**) $M_{y}$, (**f**) $M_{z}.$

Model comparison results are shown in Figure 11, where black is the true value of the verification set, light blue is the proposed two-stage decoupling model, red is linear regression, green is polynomial fitting, dark blue is random forest, and purple is BP neural network. It can be seen that the two-stage decoupling model is better than other models, especially the regression of a zero value, but the error of individual sample points is large. The error of linear regression is the largest, and it is not suitable to capture the nonlinear relationship between strain and load when multidimensional force is loaded. The error of single polynomial regression is smaller among the four contrast models, which is close to the two-stage decoupling model. The regression results of random forest for $F_{z}$, $M_{x}$, and $M_{y}$ are better, but other channels are worse. The regression error of a single BP neural network is larger for a zero value and smaller for a nonzero value.

MAPE (mean absolute percentage error) is the mean absolute percentage error. The regression accuracy of the model is evaluated by calculating the average sum of the absolute percentage error of all sample regression values and the true value.

(13) $$MAPE=\frac{1}{n}\sum_{i=1}^{n}\frac{\left|y_{i}-\hat{y_{i}}\right|}{\left|y_{i}\right|}$$

n is the total number of sample points, $y_{i}$ is the true value, and $\hat{y_{i}}$ is the regression value. The MAPEs of the five models are shown in Table 4.

In addition to decoupling errors, model complexity is also a key index to evaluate model performance. Now only the complexity calculation of the validation process is considered, and the method is as follows:

(1) Time complexity

(14) $$O{\left(A\cdot B\right)}_{pr}+O{\left(A\cdot \sum_{i}M_{i}M_{i+1}\right)}_{bp}$$

(2) Space complexity

(15) $$O{\left(A\cdot C+B\right)}_{pr}+O{\left(P+Q\right)}_{bp}$$

where the lower-right footer pr denotes polynomial regression, the footer bp denotes BP neural network, A is the number of validation set samples, B is the number of polynomial coefficients, and C is the number of polynomial variables. $M_{i}$ is the number of neurons in the ith layer (the input layer is the first layer, and the output layer is the last layer), P is the number of neural network weights, and Q is the number of biases.

The time complexity of the validation process according to the two-stage model is calculated to be O(1531530), and the space complexity is O(13363), given a sample size of 117.

A comparison of the research progress of six-component force sensor decoupling models within the last 6 years is shown in Table 5, which contains a comparison of the error, model complexity, and application scope of the force and moment channels. The assessment of model complexity in the table is more subjective due to the unavailability of model codes from other researchers.

Combined with Figure 11, Table 4 and Table 5, it is summarized below:

(1) The two-stage model based on simulation data has a mean MAPE of 6.29% in the force channel and 3.24% in the moment channel, which is not much different from the single BP neural network regression model. It indicates that the operation of applying the regression model in the subchannel combines the advantages of the two single models, and the training of the corresponding models according to different load types can reduce the error.
(2) The zero-setting operation in the classification stage largely reduces the regression error on the value of 0. However, it depends on the model performance of the deep BP neural network, which needs to be optimized in terms of the amount of data in the training set, the structure of the network, the training function, and the learning rate.
(3) In the case of mixed loading of multidimensional forces, there is a nonlinear relationship between the load value and the strain value, and it is not appropriate to use a linear model, and the polynomial regression and BP neural network are more effective in a single model.
(4) From the aspect of error, this paper targets the decoupling of multidimensional loading cases, and the model input and output are nonlinear relationships, which need to be followed up by increasing the number of samples in the training set and optimizing the model parameters to further improve the model performance. From the aspect of model complexity, the current state of the art of the research in recent years has mostly been the combination of nonlinear models, which has certain requirements for the hardware arithmetic and the amount of training data in the real-world applications. From the aspect of the application in terms of application, the two-stage model has stronger generalization and can be applied to multidimensional load decoupling.

## 5. Static Calibration Experiment and Analysis of Results

### 5.1. Experimental Scheme and Establishment of Dataset

**Figure 12:**
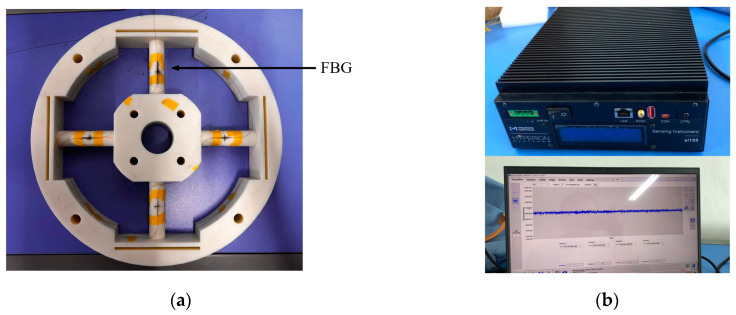

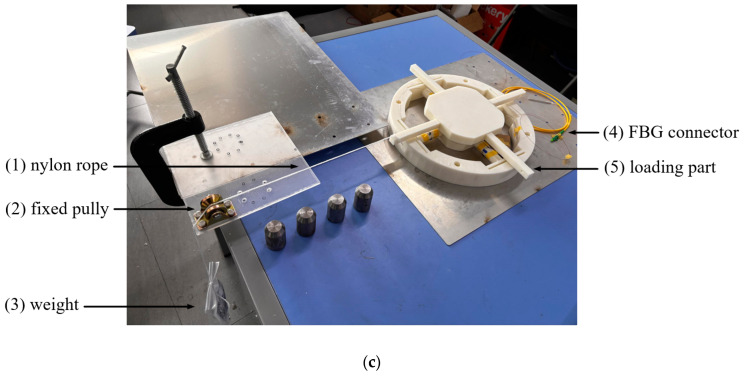
Calibration experiment: (**a**) elastomer sample, (**b**) FBG demodulator, (**c**) loading of force.

In order to further test the reasonableness and generalization of the two-stage decoupling model in real applications, static load calibration experiments of elastomers with resin materials were carried out, as shown in Figure 12, and the experimental tools mainly consisted of (1) nylon ropes, (2) fixed pulleys, (3) weights, (4) FBG joints, and (5) loading part. The temperature of the experimental environment was maintained at about 20 °C. A total of 16 FGBs were fused in series and pasted onto the elastomer surface, and after the center wavelength data of the FBGs were collected by a demodulator, the wavelength data were converted into strain data according to Equations (3) and (4), and then, the static load calibration experimental dataset was established in accordance with Table 3.

The total number of samples in the training set based on the experimental data is 384, of which 24 are single-dimensional samples, 72 are two-dimensional samples, and 96 are three-dimensional samples. The total number of samples in the validation set based on the experimental data is 41, of which 6 are single-dimensional samples, 15 are two-dimensional samples, and 20 are three-dimensional samples. It should be noted that, due to the limitation of experimental conditions, the maximum loading in the dataset is 3, which means that the experiment only verifies the model feasibility in the case of one-dimensional, two-dimensional, and three-dimensional loadings. The range of the force loading is from 2.5 N to 12.5 N, the range of the moment loading is from 750 N to 3750 N, and the forces and moments are isometric loading. Some of the loading schemes in the validation set are shown in Table 6.

### 5.2. Result of Classification Stage Based on Experimental Data

**Figure 13:**
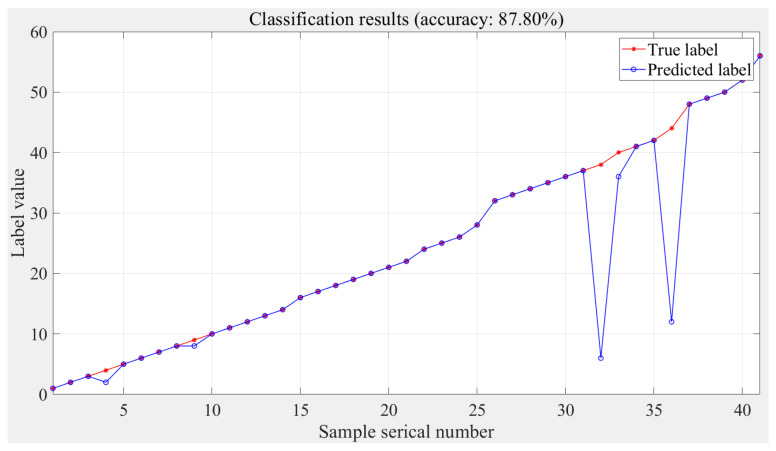
Classification stage results based on experimental data.

The prediction results of the classification stage based on the experimental data are shown in Figure 13, and the accuracy of the decoupled model is 87.80%. The labels with errors are 4, 9, 38, 40, and 44. Similar to the classification results based on the simulation data, the prediction accuracies of the two-dimensional and three-dimensional are relatively low.

### 5.3. Result of Regression Stage Based on Experimental Data

**Figure 14:**
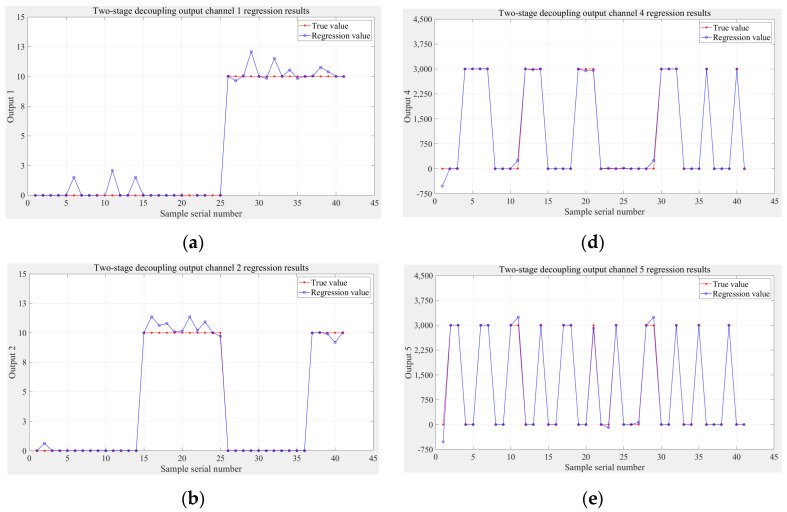

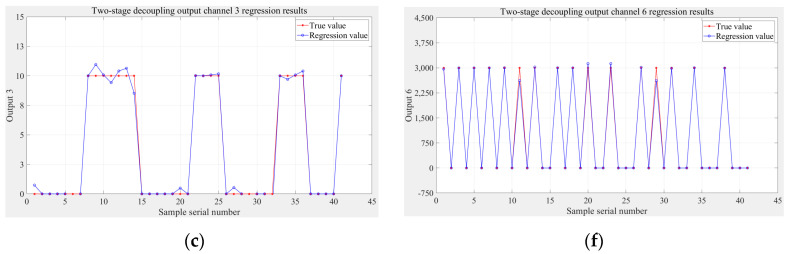
Regression stage results based on experimental data: (**a**) $F_{x}$, (**b**) $F_{y}$, (**c**) $F_{z}$, (**d**) $M_{x}$, (**e**) $M_{y}$, (**f**) $M_{z}.$

The results of the regression stage based on the experimental data are shown in Figure 14, from which it can be seen that the zero-setting operation in the two-stage model still has a significant effect on the experimental data; similar to the simulation results, the regression results of the two-stage model for the torque channel are better than those for the force channel.

Combined with Figure 10, Figure 14, Table 4, and Table 7, it is summarized below:

(1) The average MAPE of the two-stage model based on the experimental data is 5.63% for the force channel, 4.82% for the moment channel. Comparing the MAPEs of each output channel in the simulation and experiment, it can be seen that there is not much difference between the two, which indicates that it is feasible to use the two-stage decoupling model in practical engineering, and also reflects the generalization of the model.
(2) Due to the problems of funds and time, the loading schemes in the experiments are all isometric and integer multiple loading, which is inconsistent with the random loading in the simulation. If the subsequent experimental conditions are sufficiently adequate, a digital loading platform, loading parts with more optimized structure, and expansion of the experimental dataset will be used to further verify the feasibility of the two-stage decoupling model.
(3) Personally, I believe that the two-stage decoupling model can realize real-time six-dimensional force dynamic monitoring under sufficiently ideal conditions in terms of dataset and model optimization.

## 6. Conclusions

A two-stage decoupling model combining an artificial neural network and polynomial regression is proposed for the decoupling problem of a six-component force sensor in the case of multidimensional mixed loading. The elastomer structure of the sensor and the FBG arrangement scheme are designed, and a dataset based on the simulation and experimental results is constructed for verifying the feasibility of the proposed decoupling model. The validation results show that the MAPEs of the simulation and experimental results are similar, in which the decoupling model based on the experimental data has a MAPE of 5.63% for the force channel and 4.15% for the moment channel, and the performance of the model is significantly better than that of linear fitting, polynomial regression, and random forests, etc. However, there is still room for improvement in the performance of the model when compared with the one-dimensional decoupling. The above work provides ideas for the decoupling research of six-component force sensors in the field of rotor system structure monitoring.

## Figures and Tables

**Table 1:** Classification label.

Label_D	${Label\_B\_F}_{x}$	${Label\_B\_F}_{y}$	${Label\_B\_F}_{z}$	${Label\_B\_M}_{x}$	${Label\_B\_M}_{y}$	${Label\_B\_M}_{z}$
1	0	0	0	0	0	1
2	0	0	0	0	1	0
3	0	0	0	1	1	0
…	…	…	…	…	…	…
61	1	1	1	1	0	1
62	1	1	1	1	1	0
63	1	1	1	1	1	1

**Table 2:** Coefficient of term.

Term	$\mathrm{Coefficient}/10^{10}$	Term	$\mathrm{Coefficient}/10^{10}$	Term	$\mathrm{Coefficient}/10^{10}$
$p_{1}$	−0.0154	$p_{1}p_{8}$	−124.2412	$p_{5}p_{8}$	6.1983
$p_{2}$	0.0448	$p_{2}p_{3}$	−237.4281	$p_{6}p_{7}$	−11.7997
$p_{3}$	−0.1064	$p_{2}p_{4}$	320.6829	$p_{6}p_{8}$	13.1048
$p_{4}$	0.0279	$p_{2}p_{5}$	7.2262	$p_{7}p_{8}$	260.7574
$p_{5}$	0.0337	$p_{2}p_{7}$	−451.7682	${p_{1}}^{2}$	−67.8017
$p_{6}$	−0.0537	$p_{2}p_{8}$	169.8071	${p_{2}}^{2}$	−55.8811
$p_{7}$	−0.0037	$p_{3}p_{4}$	557.7507	${p_{3}}^{2}$	−95.2274
$p_{8}$	−0.0395	$p_{3}p_{5}$	−463.7950	${p_{4}}^{2}$	−510.3515
$p_{1}p_{2}$	125.5442	$p_{3}p_{6}$	407.8164	${p_{5}}^{2}$	−17.4421
$p_{1}p_{3}$	172.8450	$p_{3}p_{8}$	−81.0260	${p_{6}}^{2}$	10.6449
$p_{1}p_{4}$	−280.3908	$p_{4}p_{6}$	44.3733	${p_{7}}^{2}$	−410.6128
$p_{1}p_{5}$	96.2808	$p_{4}p_{7}$	628.2058	${p_{8}}^{2}$	−0.6020
$p_{1}p_{6}$	−102.6919	$p_{4}p_{8}$	−315.6849	c	0
$p_{1}p_{7}$	400.2304	$p_{5}p_{7}$	43.5794	—	—

**Table 3:** Dataset of simulation strain results.

Label		Force/N			Moment/N·mm			$Strain/10^{-6}$ε		
Label_D	Label_B	$F_{x}$	$F_{y}$	$F_{z}$	$M_{x}$	$M_{y}$	$M_{z}$	$p_{1}$	…	$p_{8}$
1	000001	0	0	0	0	0	100,000	43	…	171
2	000010	0	0	0	0	100,000	0	342	…	68
3	000011	0	0	0	0	123,373	941,981	512	…	1618
…	…	…	…	…	…	…	…	…	…	…
61	111101	9555	2797	3332	372,349	814,763	291,549	3602	…	918
62	111110	9918	4221	1491	864,458	466,040	624,266	2124	…	729
63	111111	9980	8450	9701	375,133	320,063	809,102	3217	…	739

**Table 4:** Comparison of MAPE based on the simulation data.

	Linear Regression (%)	Polynomial Regression (%)	Random Forest (%)	BP Neural Network (%)	Two-Stage Model (%)
$F_{x}$	33.81	10.64	27.71	26.23	7.11
$F_{y}$	38.43	9.45	32.64	17.54	5.63
$F_{z}$	33.52	12.95	15.41	24.15	6.12
Mean of force channel	35.25	11.01	25.25	22.64	6.29
$M_{x}$	22.75	8.87	17.07	6.91	4.24
$M_{y}$	19.16	9.22	12.29	5.45	3.86
$M_{z}$	41.87	15.12	24.26	5.74	1.61
Mean of moment channel	27.93	11.07	17.87	6.03	3.24

**Table 5:** Comparison of research progress.

Name of Model	Year of Publication	Mean Error of Force Channel (%)	Mean Error of Moment Channel (%)	Model Complexity	Scope of Application
Linear regression + support vector machine [18]	2018	0.87	0.87	Complicated	Single dimension
Random forest + genetic algorithm [19]	2020	0.67	0.72	Complicated	Single dimension
Polynomial regression [20]	2023	0.92	0.05	Simplex	Single dimension
BP neural network + genetic algorithm [21]	2023	0.15	0.15	Complicated	Single dimension
Two-stage model	2024	6.29	3.24	Complicated	Multiple dimension

**Table 6:** Partial validation set based on experimental data.

Label_D	$F_{x}$/N	$F_{y}$/N	$F_{z}$/N	$M_{x}$/N·mm	$M_{y}$/N·mm	$M_{z}$/N·mm
1	0	0	0	0	0	3000
2	0	0	0	0	3000	0
4	0	0	0	3000	0	0
8	0	0	10	0	0	0
16	0	10	0	0	0	0
32	10	0	0	0	0	0
3	0	0	0	0	3000	3000
24	0	10	10	0	0	0
9	0	0	10	0	0	3000
56	10	10	10	0	0	0
7	0	0	0	3000	3000	3000
25	0	10	10	0	0	3000
11	0	0	10	0	3000	3000

**Table 7:** MAPE based on experimental data.

$F_{x}$	$F_{y}$	$F_{z}$	Mean of Force Channel	$M_{x}$	$M_{y}$	$M_{z}$	Mean of Moment Channel
5.74%	6.29%	4.86%	5.63%	6.53%	2.03%	3.91%	4.15%

## Data Availability

After a while, part code of the Two-stage decoupling model and part of the dataset in this research will be posted on the GitHub account of the first author [Shuyu Wang]. The first author will regularly check the GitHub account and answer questions about this research in free time. https://github.com/HammeeerrrWang.
